# The Team Science Readiness Framework for scientific research collaboration

**DOI:** 10.3389/fpsyg.2026.1792280

**Published:** 2026-08-19

**Authors:** Molly P. Kilcullen, Ibrahim Bukhari, Mia Terkowitz, Sarah Stevens, Cyd Lacanienta, Michael Rosen

**Affiliations:** 1 School of Medicine, The Johns Hopkins University School of Medicine, Baltimore, MD United States; 2 School of Education, Johns Hopkins University School of Education, Baltimore, MD, United States

**Keywords:** framework, readiness, science teams, team science, teamwork

## Abstract

**Introduction:**

Multidisciplinary research teams need a practical structure for assessing and improving their readiness to collaborate effectively. We developed the Team Science Readiness Framework (TSRF) to address this need.

**Methods:**

Team science considerations were initially identified by team science and healthcare experts, including the authors and affiliated collaborators, and were supplemented through a literature review. The framework was iteratively refined, prioritizing practical relevance, robust empirical support, and actionable factors that could be targeted through feasible interventions for improvement or sustainment.

**Results:**

The resulting TSRF comprises tangible, evaluable, and actionable considerations for science teams and provides a structure for organizing existing tools and resources. It addresses the research process across three stages: building teams during the early phases of a research project; managing teams while executing the research plan; and sustaining, growing, or gracefully closing teams as individual projects conclude.

**Discussion:**

By emphasizing actionable and empirically supported constructs, the TSRF offers a practical approach to evaluating multidisciplinary research-team readiness and guiding interventions throughout the team lifecycle.

## Introduction

There is an abundance of existing teamwork research, frameworks, and assessments in psychology literature (Rousseau et al., 2006; Huang et al., 2023) and this team science knowledge has been applied to healthcare contexts with increasing emphasis over the last couple decades. The field of research and development has a long history of studying teamwork in research teams (Barry et al., 1999; Levi and Slem, 1995). The importance of team effectiveness in research teams became so widely recognized that an entire body of literature on the Science of Team Science (SSTS; Yu et al., 2019) arose, which focuses on exploring the behaviors and attributes of team-based collaboration in scientific teams. This field of research has rapidly developed over the last 20 years, highlighting challenges in characterizing and evaluating cross-disciplinary research initiatives (Stokols et al., 2008). Multi-level perspectives on research teams have investigated the macro (i.e., population level), meso (i.e., team level), and micro (i.e., individual level) levels of teamwork in science teams (Börner et al., 2010). However, there is a gap in the literature addressing the temporal levels of research teams (i.e., phase of research stage) and how the importance of individual team science factors may fluctuate as a function of the stage of research that teams are experiencing.

Team science readiness, or how prepared individuals and the team are for scientific collaboration, has been a point of interest since the conception of SSTS (Hall et al., 2008). Early conceptualizations of team science readiness theorized the importance of individual and team research orientations (i.e., attitudes toward collaboration), resources for collaboration, and integrating collaborative efforts across fields (Hall et al., 2008). Providing structure for collaboration is particularly important as health research initiatives often involve integrating efforts across academic institutions, care facilities, foundations, and government entities (Hall et al., 2008). There is a need for research to address the multiple levels and dimensions of readiness for multidisciplinary research teams to uncover the factors that impact the effectiveness of research projects (Hall et al., 2008; Stokols et al., 2008). The diverse readiness factors that impact teamwork in research teams have yet to be adequately studied, conceptualized, and targeted for intervention.

The current effort addresses these needs by developing and refining a Team Science Readiness Framework (TSRF) focused on the essential competencies for healthcare research teams, segmented by the phases of science team research. This framework was developed first from subject matter expert (SME) opinion and then supplemented by established team science literature. The framework provides a foundation for understanding the contextual factors that contribute to science team readiness and subsequent team effectiveness across the science team’s lifespan. This framework uniquely contributes to the existing literature on the science of team science and team readiness by addressing the temporal nature of science teams, by identifying the key team science needs at each phase of the research process. For this framework, we define the research process phases to include the team processes focused on building teams during early phases of a research project, managing teams while executing a research plan, sustaining and growing teams as specific research projects come to an end, and gracefully closing teams that are not continuing their collaboration beyond that project. While some elements of the framework (e.g., building trust and psychological safety and promoting team adaptability and resilience) are important throughout a team’s lifecycle, this temporal framework will be designed to help practitioners focus on specific team practices at critical junctures in a team’s development. Additionally, we review common challenges that research teams face during each of these stages to provide context for the importance of team science factors. This framework can be used to organize and target interventions as well as identify gaps in available tools.

Below, we review each element of the Team Science Readiness Framework (TSRF). Team science considerations were first developed from team science and healthcare experts (including the authors and affiliated collaborators) and then supplemented by reviewing the literature on teamwork, team science, the science of team science, and team and individual readiness for collaboration. Existing frameworks and relevant team science concepts were reviewed by team science and healthcare subject matter experts. These experts consolidated, integrated, and discussed each of these factors in detail, with attention being given to the factors that are particularly relevant to healthcare research teams. Additionally, emphasis was placed on incorporating factors into the TSRF that were deemed “actionable,” such that interventions could feasibly be implemented to target that factor for improvement or sustainment. These team science and healthcare subject matter experts worked together in a collaborative and iterative manner to map the chosen factors to the phases of research to address the temporal considerations for science team readiness. The resulting framework is comprised of tangible, evaluable, and actionable team science considerations for science teams, providing a needed structure to organize existing tools and resources in an actionable way. This approach prioritized practical relevance and the inclusion of constructs with robust empirical support that have been widely studied within the team science literature. This effort represents the initial phases in framework development, laying out the foundation for framework formal validation and refinement.

## Team Science Readiness Framework

**FIGURE 1:**
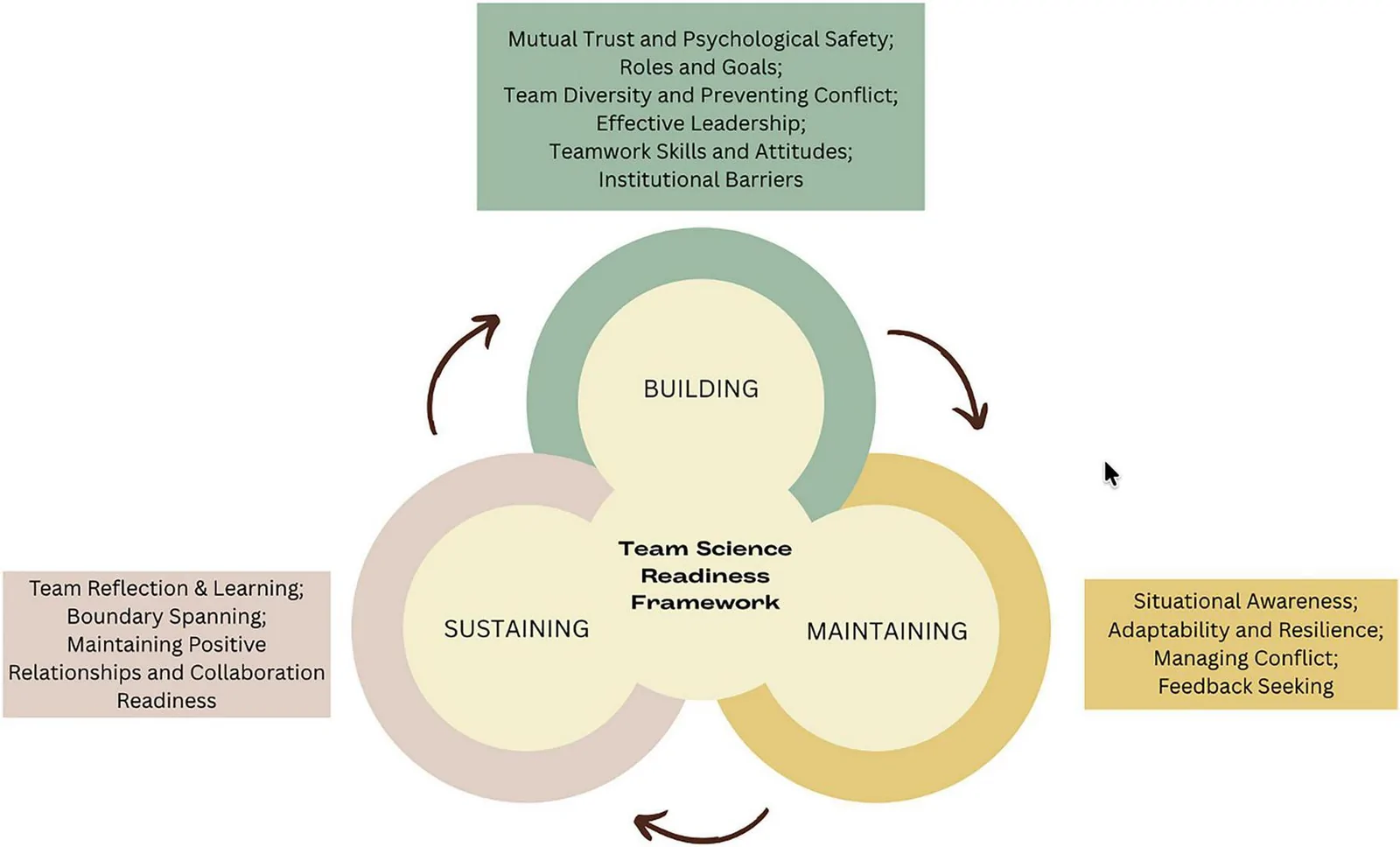
The Team Science Readiness Framework.

As previously mentioned, the TSRF breaks down the research process into three stages (See Figure 1 and Table 1). Specifically, core frameworks from the science of teams (Ilgen et al., 2005; Marks et al., 2001) were considered as they related to steps in a patient engaged research process (Mullins et al., 2012), with the guiding principle that different aspects of teamwork would be more or less important at different phases of the team’s development and the nature of the work being done. First, team members must consider the team science considerations relevant to building the project team. Teams must then transition to considering the factors relevant to maintaining effective teamwork processes. Finally, science teams gracefully close the project or sustain and grow the project team if members intend to continue working together beyond the initial scope of the project. If teams grow their project team, they will feed back into the building phase, as they integrate new team members (see Figure 1). This framework is intended to focus on the team science factors that teams and leaders can directly address. As such, factors that facilitate team effectiveness, but that were deemed outside the control of leaders and the teamwork context (e.g., institutional culture, educational training) were excluded. Additionally, this framework is intended to be a guide which teams can use to target evaluative efforts and interventions at each of the research stages. It was beyond the scope of the current work to systematically review and integrate teamwork trainings and other interventions corresponding to each consideration; however, we address this as an opportunity in the future directions section below. Team science considerations included in each phase were developed through SME input and are thoroughly supported through established team science literature. Although this framework considers phases of teamwork beyond the initial phase of building a team, the framework is so named because team readiness encompasses not only the formation and function of teams but also preparation for maintenance and effective closure. The name conveys that teams should be prepared for all stages of the lifecycle, including sustaining and closing out operations.

**TABLE 1:** The Team Readiness Framework.

Considerations	Building	Managing	Sustaining/closing
Research process	1. Soliciting topics 2. Recruiting the team 3. Applying for funding 4. Prioritizing research 5. Framing the question 6. Selecting comparators and outcomes 7. Creating a conceptual framework	8. Analyzing the research plan 9. Collecting data	10. Reviewing and interpreting results 11. Translating results 12. Disseminating findings 13. Identifying subsequent opportunities
Common research team challenges	• Lack of common vocabulary • Difficulty integrating knowledge across disciplines • Goal misalignment • Geographic dispersion	• Team member instability (i.e., permeable boundaries) • High task interdependence	• Team member instability (i.e., permeable boundaries) • Institutional competition
Key team science considerations	• Mutual trust and psychological safety • Roles and goals • Team diversity and preventing conflict • Effective leadership • Teamwork skills and attitudes • Institutional barriers	• Situational awareness • Adaptability and resilience • Managing conflict • Feedback seeking	• Team reflection and learning • Boundary spanning • Maintaining positive relationships and collaboration readiness

### Stage one: building teams

The first stage, building teams is characterized in this framework by scientific teams soliciting topics of interest, framing the research question they want to address, selecting comparators and outcomes for their study, and creating a conceptual framework. This stage focuses on building the readiness of existing teams. Common challenges that teams face during their inception include lack of common language (e.g., jargon), difficulty integrating knowledge, goal misalignment, and geographic dispersion (National Research Council, 2015; Tannenbaum et al., 2023). Team members apply their unique skills and knowledge to the team’s goals; however, team functional and educational diversity can lead to issues with jargon, which impedes knowledge integration within the team. Interinstitutional teams or teams with high diversity may also initially have misaligned or conflicting goals at the onset of the collaboration. Finally, geographic dispersion of research team members can cause issues with communication, coordination, and institutional policy misalignment. For this first stage in the research process, there are several team science considerations identified, including mutual trust and psychological safety, team roles and goals, team diversity and conflict prevention, effective leadership, teamwork skills and attitudes, and institutional barriers, all of which have an impact on science team problem-solving and innovation.

#### Mutual trust and psychological safety

When there is mutual trust, team members can be relied upon to complete their work, do what’s right, and be honest (Salas et al., 2005; Ryan, 2012; Wong and Sohal, 2002). Mutual trust among team members facilitates the development of psychological safety. Psychological safety is how safe team members feel to speak up and engage in risk taking (Edmondson, 1999). When psychological safety is present, team members have the confidence to take risks, such as brainstorming new ideas, confronting interpersonal or task-related conflict, and compromising without fear of negative repercussions (National Research Council, 2015). When there is psychological safety and voice (i.e., verbal expression of ideas, suggestions, and concerns; Erkutlu and Chafra, 2015) within a team, members feel like: (1) they can speak up without fear of retribution, (2) that their input is valued and considered, and (3) that team leaders are willing to act on team members’ concerns (Chan et al., 2003; Upenieks et al., 2010; Pack et al., 2022). Mutual trust, psychological safety, and voice are essential for science teams, as evidence indicates that they contribute to team innovation and creative problem-solving (Frazier et al., 2017; Oppert et al., 2022; Zhu et al., 2022).

#### Team roles and goals

In the initial phase of scientific team collaboration, teams establish mutually agreed upon team member roles and responsibilities, as well as shared team goals and vision. Not only is it essential for team members to clearly delineate their roles and responsibilities, but team leaders have distinct functions within the team that should be clearly established (Morgeson et al., 2010). Common leadership responsibilities extend across all stages of the research process and include composing the team, providing feedback, monitoring the team, boundary spanning, and conflict management (Morgeson et al., 2010). If team member or leader roles are unclear or implicit, instead of explicit, it can lead to a lack of accountability and issues with task coordination. It’s important during this phase for teams to implement a deliberate process for creating a shared vision for the team and for clarifying and aligning goals (Brasier et al., 2023). Identifying gaps in team member perspectives or expertise is essential in the initial phases of building science teams to enhance team creativity and innovation, as goal clarity impacts team processes and subsequent performance (Peralta et al., 2015). When members ensure that they are able to identify the expertise of other team members clearly, they expedite team problem-solving.

#### Team diversity

During the creation of scientific teams, functional diversity of the team (e.g., educational degrees, specialties, backgrounds, cultures), as well as surface-level diversity (e.g, race, gender) are important to consider when composing and assessing teams. High diversity in research teams often poses a challenge with establishing common vocabulary, leading to communication issues amongst the team (National Research Council, 2015). Task-related diversity, but not bio-demographic (i.e., surface-level) diversity, has a positive impact on team outcomes (Horwitz and Horwitz, 2007). Task-related diversity refers to the team’s individual attributes, such as functional expertise, education, and organizational tenure (Horwitz and Horwitz, 2007). While the relationship is complex, there is evidence to indicate that team diversity impacts research performance (Barjak and Robinson, 2008; Saá-Pérez et al., 2017). To effectively manage scientific teams, existing evidence indicates that leaders must consider institutional diversity (i.e., how many team members are within the same organization), educational diversity (e.g., Ph.D., M.A., M.D.), and discipline diversity (e.g., surgery, pediatrics, engineering, psychology) when recruiting new members (Saá-Pérez et al., 2017).

#### Conflict prevention

There are three distinct types of team conflict, which are task conflict (i.e., different perspectives on what work needs to be done), process conflict (i.e., different ideas about how the work should be done), and relationship conflict (i.e., interpersonal tensions; O’Neill et al., 2013). Team diversity might result in conflicting norms and expectations for the work, which could ultimately lead to any of the three types of team conflict. Indeed, cultural diversity is associated with task and relationship conflict but also related to increased creativity and satisfaction (Stahl et al., 2010). Effective conflict management within teams can help mitigate the impact of conflict on team performance. Effective conflict management for research teams involves proactively identifying sources of conflict, such as authorship, ownership of data, and intellectual rights early in the collaboration process. After identifying potential conflict points, effective teams establish norms for addressing those conflict areas before they can disrupt team problem-solving and innovation. Indeed, team norm setting and development of guidelines for how they want to handle conflict (e.g., established conflict management strategies) are a core necessity for effective teamwork (Salas et al., 2015).

#### Effective leadership

Effective scientific teams have leaders that promote a supportive environment for collaboration (Wu and Cormican, 2021). Additionally, research teams should have a shared leadership style such that leadership responsibilities are assumed by team members that have the appropriate skills and experience for the task in question (Wu and Cormican, 2021). Shared leadership, or when team members collectively lead the team instead of having a sole designated leader (Carson et al., 2007), can be especially useful for teams with complex workflows (Kozlowski et al., 2016). Shared leadership has been shown to be correlated with higher team effectiveness (Ensley et al., 2006; Wang et al., 2014; Serban and Roberts, 2016). Also, meta-analytic evidence shows that several different leadership styles, such as empowering leadership (i.e., enabling team members by allocating responsibilities and encouraging self-management; Burke et al., 2006; Cheong et al., 2019) and supportive leadership (i.e., leadership focusing on providing access to resources; Lee et al., 2020), can promote creative and innovative team performance (Lee et al., 2020). Beyond leadership styles, teams should consider how their leaders can capitalize on opportunities outside the immediate team environment. Although this framework focuses on actions within individual research teams, team leaders frequently occupy positions that extend beyond the team itself. These broader roles provide opportunities to influence organizational readiness, support the development of future researchers, and foster conditions that facilitate effective team science.

#### Teamwork skills and attitudes

Meta-analytic evidence supports that team orientation, an individual or team’s orientation toward prioritizing team goals over individual goals, is correlated with team innovation and performance (Kilcullen et al., 2023). When team orientation is high, individuals within the team are more likely to engage in team back-up behaviors (Kilcullen et al., 2023), which subsequently improves team performance. Effective teams have team members that believe that their team can effectively tackle their shared goal, value being a part of the team, and possess the right interpersonal skills to collaborate with each other. Existing evidence indicates that collective efficacy beliefs, team cohesion (particularly task cohesion), and back-up behaviors (i.e., proactive and reactive assistance from team members to ensure team functioning and completion of team tasks; Marks et al., 2001) promote team performance (Grossman et al., 2022; Li et al., 2020; Porter et al., 2011). When recruiting for new members for a scientific team, consideration of teamwork skills and attitudes is warranted. As the initial phase of team building develops, effective science teams monitor their efficacy, cohesion, and team-based behaviors. Additionally, functioning science teams implement a deliberate process for engaging in back-up behaviors, such that team members step in to help when needed, even when it isn’t their responsibility. While teamwork skills and attitudes are important to consider at team selection, they can also be developed over time. Teamwork skills can be enhanced in team members and attitudes slowly influenced over time from targeted trainings and interventions (Lacerenza et al., 2018; McEwan et al., 2017). Therefore, while included in the building phase, teamwork skills and attitudes are applicable to the maintaining and sustaining portions of the team life cycle.

#### Institutional barriers

Institutional constraints have been identified in the team science literature as important contextual factors that can influence team effectiveness (Hall et al., 2008; Stokols et al., 2008). During initial formation of research collaborations, the institutional barriers to the team’s work have a major impact on team performance. Institutional barriers could include policies that impede teamwork or a lack of support or resources from the institution (e.g., interpersonal skills training, technological systems for collaboration). Collaborative technology, such as data sharing platforms and communication technology facilitate teamwork (Zhang et al., 2011). Additionally, effective administrative processes for sharing data across team member organizations (e.g., data use agreements) and interoperable platforms for sharing and analyzing data enable research team functioning. They need not only access to communication technology, but also the knowledge and skills to utilize the available technology effectively. While institutional barriers are challenging to address, acknowledging constraints is essential for anticipating barriers to team problem-solving and goal attainment.

### Stage two: managing teams

After the initial phase of building a science team, the team then transitions to teamwork maintenance. This second research phase is managing teams, which involves the study team analyzing the research plan and collecting data for the research study. Challenges that teams experience during the managing teams phase include team member instability, high task interdependence, and lack of leadership accountability (National Research Council, 2015; Tannenbaum et al., 2023). Many large projects involve several phases of research, in which team membership may change expertise needs fluctuate across project phases. Integrating interdependent contributions of team members, particularly for interinstitutional or interdisciplinary teams, can be challenging and present opportunities for conflict. Finally, the second stage a common teamwork challenge is leadership failing to effectively promote collaboration, adaptation, and conflict management within the team. For this stage, the key team science considerations identified include promoting situational awareness, team adaptability and resilience, managing conflict, and encouraging feedback seeking.

#### Team situational awareness

There is an abundance of literature in healthcare on the importance of team situational awareness (Jonsson et al., 2021; Weller et al., 2024; Wright and Endsley, 2017). Team situational awareness is the dynamic process involving members perceiving cues within their environment, understanding the meaning behind those cues, and being able to predict how the situation might evolve over time (Weller et al., 2024). Maintaining situational awareness of the team’s progress toward shared goals and maintaining goal clarity as the team works through its project is of the upmost importance. Team members should always know where the team is regarding goal progress and what will come next to achieve the team’s goals (Sætrevik, 2013). Maintaining situational awareness is connected with the next team science consideration in this research stage, “adaptability,” as it involves monitoring the team’s progress toward its objectives, enabling team members to adjust as needed (Sætrevik, 2013).

#### Team adaptability and resilience

The second team science consideration for managing teams is the team’s ability to adapt to changing circumstances and their resilience to in facing obstacles. Teams need to have the ability to adapt well to change and remain alert to respond to early signs of issues among team members (McEwen and Boyd, 2018). Scientific teams should implement deliberate processes for encouraging adaptive behaviors to function effectively in situations with changing conditions, demands, and needs. This is particularly important for teams with permeable boundaries, such that there is ongoing membership change causing disruption to team functioning (National Research Council, 2015). Behavioral markers for team adaptability relate to other team science considerations discussed, including situational awareness, team communication, role differentiation (i.e., role clarity), and affect management (Rosen et al., 2011), highlighting the importance of those pre-existing conditions and the ability to adapt those conditions as context demands. Team adaptability often contributes toward team resilience, as adverse events can disrupt team activities, prompting teams to change their processes (Hartwig et al., 2020). Team resilience refers to their capacity to adapt and recover from setbacks and adversity while maintaining team functioning and performance (Alliger et al., 2015). Team resilience has been found to be indirectly related to team performance, through creative problem-solving (Carmeli et al., 2021). To enhance resilience and promote problem-solving, science teams should utilize existing tools to develop team adaptability (Rosen et al., 2011; Van der Beek and Schraagen, 2015).

#### Conflict management

While teams should consider conflict prevention during the building phase, conflict management must remain a consideration throughout the managing teams phase. Existing evidence indicates that the relationship between conflict and team performance can be complicated (De Wit et al., 2012; O’Neill et al., 2013). While process and relationship conflicts have been shown to have negative impacts on team performance, task conflict has been shown to have beneficial effects on performance (O’Neill et al., 2013). Task conflict refers to disagreements amongst the team regarding work processes or goals, which can promote constructive discussion (Jehn, 1995). This relationship is particularly true for decision-making teams, suggesting that task conflict can contribute toward the development of innovative solutions. However, it is important for the team to maintain a psychologically safe environment to approach the task conflict in a constructive manner. Scientific teams need to have the processes in place to manage both interpersonal and task conflict within the team. While teams should have already set norms and guidelines for how they want to handle conflict during the building phase, it is important for teams in the managing phase to stick to those norms or adapt them as needed as a team (Salas et al., 2015). As task conflict can enhance innovation, science team members should encourage open discussions and conflicting opinions but have the processes in place to resolve disagreements and promote healthy interpersonal relationships.

#### Feedback seeking

The final team science consideration for the second phase of the research process, “managing teams,” is ensuring that teams are actively engaging in communicating and seeking feedback. Scientific teams should implement a deliberate process for seeking feedback from other team members (Hall et al., 2018) and ensure that team members act on feedback relating to team performance (McEwen and Boyd, 2018). While the direct impact that feedback seeking has on team performance is not clear (Anseel et al., 2015; Ashford et al., 2016), there is some meta-analytic evidence that suggests that feedback orientation is related to work performance (Katz et al., 2023). Additionally, there are implications for feedback seeking and adaptability. Feedback seeking for self-correction is a behavioral marker of team adaptability (Rosen et al., 2011) and science teams should actively engage in feedback seeking to monitor their team state (i.e., dynamic states in teams that are affective, cognitive, or motivational; Marks et al., 2001), identify any potential conflicts, and adapt behaviors or processes to facilitate resilience.

### Stage three: sustaining and growing teams or closing projects gracefully

The final phase of the research cycle is sustaining and growing teams or closing projects gracefully. This phase involves the scientific team reviewing and interpreting the results of their study, translating those results, and disseminating the findings. The team either sustains their scientific team by pursuing the next research interest or gracefully ends the collaboration in a manner that leaves the door open for future partnership. Sustaining collaborations is a key component of readiness for teamwork (Lotrecchiano et al., 2026) and can help ease the transition back to the first phase (i.e., building teams) if at least some of the team members have experience previously working together. Common challenges that teams face during this stage include team member instability and institutional competition (National Research Council, 2015; Tannenbaum et al., 2023). As projects end, teams experience a period of instability in which team membership fluctuates as a function of changing research needs, which can impede team learning and collaboration readiness. Additionally, teams may experience issues with competition, especially with recent issues with diminishing pool of federal funding and other resources. The team science considerations for this phase include supporting team reflection and learning, enabling boundary spanning, and maintaining positive relationships and collaboration readiness.

#### Team reflection and learning

The first team science consideration for this phase is enhancing team reflection and learning. Teams should regularly take time to improve work processes, discuss ways to learn from mistakes, and engage in corrective action when errors are made (Chan et al., 2003). In an ideal situation, someone on the team should ensure that the team makes time to reflect on its own work processes (Chan et al., 2003). Encouraging open dialogue creates an environment where team members can share insights about their collaboration experiences, thus fostering continuous learning and adaptation (Senge, 1990). While a certain level of team reflection and learning takes place during the managing phase through feedback seeking and adaptation, this typically takes place in an informal, *ad hoc* manner. During the sustaining and growing phase, science teams should have formal, established points for team reflection to occur, during which teams can highlight lessons learned and identify what norms, roles, and goals should be revised within the team moving forward.

When striving to sustain and grow research teams, it is essential to support team vitality, which refers to the overall health and functionality of the team. Evaluating whether team members are working together effectively as a high-functioning team is paramount. To promote team vitality, it is important for teams to evaluate their functioning regularly through reflective practices–such as team assessments and feedback sessions–that can aid in identifying strengths and areas for improvement (Upenieks et al., 2010).

#### Boundary spanning

Scientific teams should enable team boundary spanning, so team leaders and team members are effective at branching outside of the team for resources and expertise. They should investigate whether individuals from other institutions are working on similar projects, and if resources from external sources are needed or available (Ancona and Caldwell, 1992). Relating back to team learning, teams can engage in boundary spanning by inviting people from outside the team to present new information and participate in scientific discussion (Chan et al., 2003). Inter-team collaboration can bridge knowledge gaps and promote a more holistic approach to research initiatives (Börner et al., 2010). Effective collaboration between teams can lead to greater creativity and better outcomes, particularly when teams utilize diverse perspectives and expertise (Wang et al., 2019).

#### Positive relationships and collaboration readiness

Finally, when working toward sustaining the research team or ending the project team gracefully, teams should work to maintain positive relationships and collaboration readiness. Individual members should devote time to understanding how other disciplines can contribute to the team’s current or future work and continue to interact with colleagues from other disciplines (Hall et al., 2008). Additionally, it is beneficial for team members to be knowledgeable that the benefit of collaboration with people from different disciplines outweighs the potential inconveniences of that collaborative work (Hall et al., 2008).

### Limitations

While the framework is rooted in subject matter expert opinions and the existing literature on team science, it has not undergone content validation yet. There is extensive evidence to support the importance of each of the components of the framework in teams; however, less is known about the generalizability to healthcare research team contexts. Validation efforts with research are needed before resources and quality improvement efforts are targeted. Additionally, while a literature review was conducted to supplement SME input with existing evidence, the review was not systematic, which may have potentially resulted in the exclusion of relevant team science concepts. Similarly, while the vast majority of team science considerations outlined within this framework are applicable to various team contexts, certain aspects may pose challenges in generalizing this framework beyond research and research teams. Moreover, the comprehensive range of team science considerations included in the framework may lead to complexities and potential burdens for teams when navigating the corresponding assessment and toolkit.

### Future directions

Looking toward the future, efforts should be threefold. Initially, a validation effort should be conducted for the TSRF. This can be accomplished by established group consensus methods, such as a Delphi study. Responses from subject matter experts can be used to refine and validate the framework. Second, a corresponding teamwork readiness assessment tool can be developed, which could build on the established TSRF competencies. This tool could be used to pinpoint risk points for each competency domain and provide teams with valuable self-reflection prompts. It could be designed to serve as an efficient self-assessment, it could facilitate team members in identifying their strengths and weaknesses while fostering discussions aimed at improving collaboration processes.

Finally, future research and development should focus on providing support and resources to assist teams in developing the competencies within their specific contexts. To effectively develop these competencies, researchers and practitioners can focus on three key areas. Firstly, formal learning sessions can be developed to prioritize the competencies necessary for building teams during the early phases of research efforts, including virtual workshops and formal sessions. Secondly, informal learning and group coaching can be utilized to provide additional tools for managing, sustaining, and growing teams. Additionally, future work can create practical tools to support essential team science practices, ensuring that resources are accessible beyond the workshops. A systematic review of existing team science training programs and interventions, categorized under each of the team science considerations presented in our framework is an opportunity for future work. Lastly, the TSRF could be validated for applications outside of healthcare research contexts. Numerous included competencies in the framework are applicable to other science and nonscience teams.

## Conclusion

This framework of team science considerations mapped to the stages of the research process begins to address the need for understanding scientific teams in healthcare. Future work will include the development of the teamwork readiness assessment tool for teams to identify their strengths and weaknesses. Additionally, we plan to provide support and resources for teams to develop these competencies within their own contexts. The goal is to integrate a series of tools to target all competencies and provide a broad range of resources for teams that fit their needs. Researchers, particularly those engaged in team science, can utilized this framework to further their own efforts investigating the dynamic aspects of clinician and research teamwork. While this framework was specifically developed for healthcare research teams, many of the components of this framework have been shown in the team science literature to be important across team contexts.

## Data Availability

The raw data supporting the conclusions of this article can be made available by the authors upon request, without undue reservation.
